# W. Alwyn Lishman, MD, FRCP, FRCPsych (Hon)

**DOI:** 10.1192/bjb.2021.12

**Published:** 2021-08

**Authors:** Maria Ron

**Emeritus Professor of Neuropsychiatry, Institute of Psychiatry, Psychology and Neuroscience, King's College, London, UK**

**Figure:**
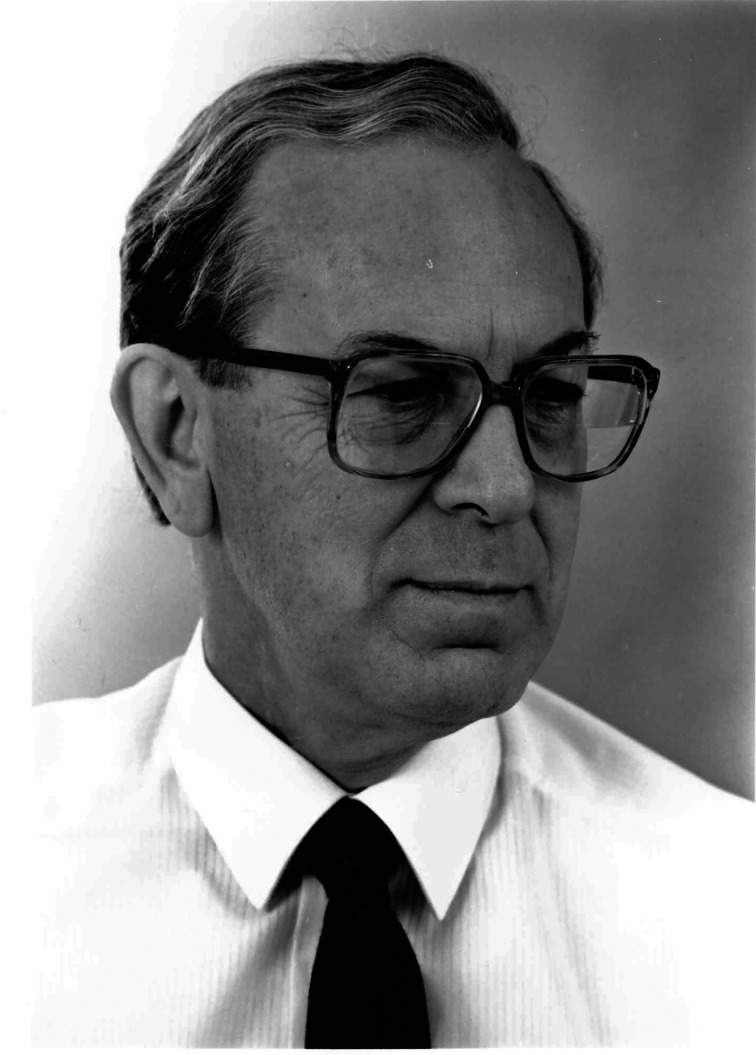

Alwyn Lishman, who died on 24 January 2021, aged 89, was the first Professor of Neuropsychiatry to be appointed in the UK. He was the co-founder and Honorary Life President of the British Neuropsychiatry Association (BNPA). In 2016, when addressing the BNPA, Alwyn said that he wanted to be remembered for what he did, in terms ‘expressed concisely’, thus setting a challenge for those of us who now wish to celebrate his exceptional contribution to psychiatry. His famous book *Organic Psychiatry: The Psychological Consequences of Cerebral Disorder*,^1^ first published in 1978, set an example of conciseness and clarity in medical writing while at the same time single-handedly defining the discipline of neuropsychiatry. Alwyn considered this book to be his most important legacy; two further single author editions followed in 1987 and 1997. At the time this book was published, neuropsychiatry was a small specialist interest. Now, thanks considerably to his influence, it is a major component of psychiatric research and practice.

Alwyn saw neuropsychiatry as much more than an admixture of neurology and psychiatry, as involving the understanding of brain mechanisms in relation to mental disorder, and benefiting from advances in neuropsychology, biochemistry, genetics, imaging and many other disciplines. For him, neuropsychiatry could equally apply to the study of schizophrenia, affective disorders or traumatic brain injury, in fact to the whole of psychiatry. By defining neuropsychiatry in this way, he was redressing the balance between brain and mind that had been polarised by the influence of psychoanalytic theory.^2^

His research was wide-ranging. Memory was one of his early interests. In what he called ‘a crude approximation to testing Freud's theory of repression’, he explored the effect of the hedonic tone of the material to be remembered, finding that, while normal people recalled pleasant material better, the pattern was reversed in depressed patients. Later his emphasis was to exploit brain imaging in relation to psychiatry, and studies of brain damage in alcoholism and psychosis of epilepsy followed.

Alwyn Lishman was born in Houghton-le-Spring, County Durham, on the 16 May 1931. His father, George Lishman, had been a prisoner during the First World War and, when his poor health prevented him from becoming a doctor, he ran the family business, a tallow chandlery that supplied candles for mining and shipbuilding. His mother Madge (née Young) was a teacher, described by Lishman as something of a dragon who locked him up with his sister Valerie until they finished their homework without any mistakes. Alwyn's innate perseverance and attention to detail must have been greatly enhanced by this strict upbringing.

For somebody who was a master of words it is perhaps surprising to know that he could read music before he could read words. He assiduously played the piano from the age of 5 and wanted to be a musician but pressure from his father finally persuaded him to go to Birmingham University to study medicine. His interest in the relationship between brain and behaviour was sparked by his time working with Solly Zuckerman while doing an intercalated degree in Anatomy and Physiology. He qualified in 1956.

After his house jobs, he spent his National Service practising neurology. He was posted to the Army head injury hospital outside Oxford, where he was mentored by Ritchie Russell. Neurology attracted Lishman for its clinical precision, but the lack of effective treatments and his wish to help his patients made him consider other options and he followed Bob Cawley, his great friend from medical school, to the Maudsley Hospital in London in 1960. He said that it took a certain degree of unworldliness to make the move to a much less prestigious discipline.

He described the Maudsley in the 1960s as extremely social and great fun and he built lifelong friendships there. Jim Birley, Griffith Edwards and Mike Rutter were contemporaries. He thrived under the abrasive but kind influence of Aubrey Lewis and his doubts about psychiatry quickly evaporated when he realised that thinking at the Maudsley was as rigorous as any. He wrote his doctoral thesis on ‘Psychiatric disability in soldiers with penetrating head injuries’, using the Oxford register set up by Ritchie Russell. At a time when there was little interest in brain injury, Lishman's insights into the behavioural and psychiatric consequences of frontal and left temporal injuries were stunning.

He was appointed to his first consultant job at the National Hospital (Queen Square) in London in 1966. His clinical work there fuelled his interest in neuropsychiatry, and he cherished his interactions with neuropsychologists such as Oliver Zangwill and Elizabeth Warrington, but the dismissive attitude of some neurologists towards psychiatry prompted him to return to the Maudsley in 1967 as consultant psychiatrist. In 1979 he was appointed to the first Chair of Neuropsychiatry in the UK, at the Institute of Psychiatry. In 1987, with Jonathan Bird, he co-founded the BNPA, the first, but not the last, neuropsychiatric association in the world and still a thriving forum that welcomes people from different disciplines. He was made an Honorary Fellow of the Royal College of Psychiatrists in 1999.

Alwyn was an exceptional mentor, and he was proud that several of his trainees went on to hold chairs of neuropsychiatry. For me, the debt of gratitude is immense. When I arrived at the Maudsley in 1971 we were assigned a ‘moral tutor’ and Alwyn Lishman was mine. This led to fruitful years of research and, more importantly, we became close friends and I became part of his family. Alwyn, an accomplished musician, was generous enough to be my accompanist when I, a tone-deaf late starter, misguidedly attempted to take my clarinet grades. To my surprise he was even more nervous than I was. We tacitly agreed not to repeat the experience.

In 1966 he married Marjorie Loud, a psychiatric social worker he met while training at the Maudsley, and they had two children, Victoria and William. He continued to play the piano and the organ and built his own harpsichord. He retired early in 1993 to look after Marjorie, who had developed a brain tumour. Marjorie died in 2000. Alwyn's last few years were marred by the ravages of dementia. He is survived by his two children and two grandchildren.
